# A cautionary tale: university institutes of public health must “walk the talk”

**DOI:** 10.17269/s41997-023-00815-z

**Published:** 2023-08-29

**Authors:** Rebecca Haines-Saah, Lindsay McLaren

**Affiliations:** Department of Community Health Sciences, University of Calgary, Calgary, AB Canada

Public health is, and has long been, defined as the art and science of promoting health and preventing illness through organized efforts of society. This definition and scope convey certain elements and values, which distinguish public health from other fields of scholarship and practice (CPHA, 2017). These include population-level thinking, a commitment to health equity and its foundations in social justice, and an upstream focus on root causes of inequities, which are embedded within political and economic systems.

Important work in recent years has highlighted the weakening of these core elements of public health, in Canada and elsewhere (Yong, 2021). Hancock et al. (2020) and McLaren and Hennessy (2020), for example, highlight how the COVID-19 pandemic, despite bringing public health to the forefront, has reinforced a narrow, medicalized version that largely neglects decades of scholarship on intersecting social and ecological determinants of health that shape population well-being in highly inequitable ways.

In the light of these trends, one might expect—or hope—that public health institutes within public universities would serve as a bastion. As institutions whose role is to build and empower informed and critically engaged citizens, a public health institute within a public university should provide a space and platform to actively push back against harmful trends that are eroding the foundations that support the core values and actions within our field.

Unfortunately, that is not always the case, as we illustrate with reference to our own experience with a university institute for public health. Through individual and joint reflection, we identified two interrelated issues that offer a cautionary tale with respect to the role and responsibility of universities in our field, and which constitute a call to action for all of our institutions to “walk the talk” of public health.

The first issue is a passive failure to protect what public health is and is not. The core elements of public health—thinking about health at the population level and how it is shaped by the conditions in which we are born, grow, live, work, and age—require going against the grain of dominant mainstream discourses about health, and corresponding methods, which typically privilege biomedical and behavioural explanations devoid of context and theory.

In our setting, the ability to protect and strengthen these ideals was halted nearly from the start by a “big tent” mentality, where everyone was welcome and—by extension—everyone was an expert in public health, regardless of their actual expertise or work.

On the surface, it is hard to argue with the ostensibly inclusive “big tent” mentality. But it is highly problematic, and in fact highly exclusive, when it is accompanied by a lack of reflexivity and humility around what kind of work, by whom, rises to the top and why.

The “big tent” welcomes, and in fact privileges, downstream and depoliticized work that is usually focused within the well-defined contours of our medical care systems. Such work naturally accrues more quickly and is more easily funded. In an unquestioned neoliberal context that rewards achievements of volume by individuals, these dynamics only serve to weaken and dilute what is strong and unique about our field. When academic institutes fail to explicitly prioritize and challenge the structural sources of inequities, they become complicit in the longstanding problem of downstream drift (Baum & Fisher, 2014). A decision to intentionally conflate public health with “better health care” may widen the scope of research, but it dilutes the essence of what is unique and valuable to our field.

A second issue concerns values. Public health’s longstanding commitment to social justice means that an institute, or any entity, that claims to be about “public health” must contend with values and work towards shared value commitments.

Rather than there being an illumination of, or a working towards, stated value commitments of public health, what we experienced was that at critical moments the institute chose to repeatedly sidestep the issue and instead focus on “scientific excellence,” as though the two could be separated. A depoliticized version of “scientific excellence” means that the institute will not challenge nor take a public stand against harmful government policies, which are seen as “advocacy.”

The problems with this stance are twofold and can be illustrated with reference to one of the worst public health emergencies Alberta, and Canada, has ever seen—the crisis in deaths from unregulated, toxic drugs. First, under the guise of promoting “research excellence” it leads to inappropriately equal elevation of research underpinned by vastly different—and in fact incompatible—ideological perspectives, including individualized, reductive addiction medicine on the one hand, and radical harm reduction on the other.

Second, it leads to a heartbreaking missed opportunity to mobilize for the public good. Imagine the power that a 500 + member academic institute could have on devastatingly harmful government policy such as closure of supervised consumption sites (Livingston, 2021) if, rather than remaining silent, it came together to support a social justice–oriented and community-driven response, which should be at the core of any public health approach.

Perhaps illustrative of the extent to which values can be relegated to the periphery is the dynamics accompanying a growing reliance on philanthropic funding, frequently taking the form of a private philanthropic transaction connected to known health-damaging sectors, including the fossil fuel industry (Carroll, 2021; CPHA, 2015). In our experience, this was deemed acceptable, and not a subject for broader consultation with membership, because it permitted support of “research excellence.”

A recent piece by Kothari and Smith (2022) describes two poles, with a gradient in between, to characterize the ways in which public health engages with politics. At one pole, public health is viewed as apolitical and purposefully divorced from thinking about or engaging with politics. At the other, public health is about the public’s health—the contexts in which people are born, grow, live, work, and age—and the massive inequities of power and money that shape structural inequities in health-damaging conditions.

While the first pole clearly exists (with our experience providing one of many examples), we must stop giving it equal airtime as though it were a tenable version of public health. A depoliticized public health is false, damaging, and increasingly unjustifiable in the context of deepening inequities visible in Canada’s unregulated drug death emergency and worsening climate disaster. Public health is not, and never can be, value neutral. Any institute that does not explicitly recognize and embrace that is doing harm, to the field and to the public’s health.

Our institute’s failure to uphold these elements and values led us, both PhDs in public health who have devoted our careers to the field, and who have many important public health roles, relationships, and contributions between us, to leave.

Informed by our cautionary tale, we call on public health communities across our institutions (including universities, government, and funders) to do what it takes to “walk the talk” of public health, so that we can come together to defend the best of our field.

Rebecca Haines-Saah, Associate Professor, University of Calgary

Lindsay McLaren, Professor, University of Calgary; Senior Editor, CJPH

## Éditorial

La santé publique est définie depuis longtemps comme étant l’art et la science de promouvoir la santé et de prévenir les maladies au moyen d’une action sociétale organisée. Cette définition et ce cadre évoquent des éléments et des valeurs qui distinguent la santé publique d’autres domaines d’érudition et de pratique (CPHA, 2017). Ce sont le raisonnement populationnel, l’engagement en faveur de l’équité en santé et de ses bases dans la justice sociale, et l’intérêt en amont pour les causes fondamentales des iniquités, lesquelles sont ancrées dans les systèmes politiques et économiques.

D’importants travaux ces dernières années ont souligné l’affaiblissement de ces éléments centraux de la santé publique, au Canada et ailleurs dans le monde (Yong, 2021). Hancock et collègues (2020) et McLaren et Hennessy (2020), par exemple, ont montré que la pandémie de COVID-19, bien qu’elle ait mis la santé publique au premier plan, a renforcé une interprétation étroite et médicalisée qui néglige dans une large mesure des décennies d’érudition sur les déterminants sociaux et écologiques croisés de la santé qui structurent très inéquitablement le bien-être des populations.

À la lumière de ces tendances, on s’attendrait à ce que les instituts de santé publique des universités publiques servent de bastions. Un institut de santé publique au sein d’une université publique, dont le rôle est de former et d’habiliter des citoyennes et des citoyens informés, engagés et critiques, devrait offrir un espace et une plateforme pour repousser activement les tendances nocives qui érodent les bases des valeurs et des actions fondamentales de notre domaine.

Malheureusement, ce n’est pas toujours le cas, comme en témoigne notre propre expérience dans un institut universitaire en santé publique. À la faveur d’une réflexion individuelle et collective, nous avons cerné deux problèmes apparentés qui offrent une mise en garde en ce qui a trait au rôle et à la responsabilité des universités de notre domaine, et qui constituent un appel à l’action à tous nos établissements pour qu’ils « joignent le geste à la parole » en santé publique.

Le premier problème est une omission passive de protéger ce qu’est la santé publique et ce qu’elle n’est pas. Les éléments centraux de la santé publique – réfléchir à la santé des populations et à la façon dont elle est structurée par les conditions dans lesquelles nous naissons, grandissons, vivons, travaillons et vieillissons – nécessitent d’aller à l’encontre du discours dominant sur la santé (et les méthodes correspondantes), qui privilégie d’ordinaire les explications biomédicales et comportementales sans contexte ni théorie.

Dans notre milieu, la capacité de protéger et de renforcer ces idéaux a été stoppée presque dès le début par une mentalité de « grand chapiteau » où tout le monde est bienvenu et, par extension, où n’importe qui est spécialiste en santé publique, peu importe son expertise ou son travail réel.

En surface, il est difficile de contester cette mentalité de « grand chapiteau » en apparence inclusive. Mais elle est très problématique, et en fait très exclusive, lorsqu’elle s’accompagne d’un manque de réflexivité et d’humilité à l’égard du genre de travail qui connaît du succès, des personnes qui l’accomplissent, et des raisons de cet état de choses.

Le « grand chapiteau » apprécie, et même privilégie, un travail en aval dépolitisé qui ne sort habituellement pas des ornières bien définies de nos systèmes de soins médicaux. De par sa nature, ce genre de travail est capitalisé plus rapidement et trouve plus facilement des bailleurs de fonds. Dans un contexte néolibéral incontesté qui récompense les réalisations quantitatives des particuliers, cette dynamique ne fait qu’affaiblir et diluer ce qui fait la force et l’originalité de notre domaine. Quand les instituts universitaires ne se concentrent pas explicitement sur les sources structurelles des iniquités et ne les remettent pas en cause, ils deviennent complices d’un problème de longue date: la dérive vers l’aval (Baum & Fisher, 2014). La décision de faire intentionnellement un amalgame entre la santé publique et « de meilleurs soins de santé » peut élargir la portée de la recherche, mais elle dilue l’essence de ce qui est original et précieux dans notre domaine.

Le deuxième problème concerne les valeurs. L’engagement de longue date de la santé publique envers la justice sociale signifie qu’un institut, ou toute autre entité, qui prétend traiter de la « santé publique » doit compter avec les valeurs de la santé publique et travailler à des engagements moraux communs.

D’après notre expérience, au lieu d’éclairer les engagements moraux déclarés de la santé publique ou d’y travailler, aux moments critiques notre institut a choisi à maintes reprises d’éluder la question et de se concentrer plutôt sur « l’excellence scientifique », comme si les deux étaient dissociables. Une version dépolitisée de « l’excellence scientifique » signifie que l’institut ne contestera pas et ne prendra pas publiquement position contre des politiques gouvernementales nocives, ce qui serait selon lui des gestes de « plaidoyer ».

Le problème avec cette position est double; nous pouvons l’illustrer par l’une des pires urgences sanitaires à survenir en Alberta (et au Canada) : la crise des décès causés par les médicaments toxiques non réglementés. Premièrement, sous prétexte de promouvoir « l’excellence en recherche », cela conduit à mettre sur un pied d’égalité des travaux étayés par des perspectives idéologiques très différentes – et en fait incompatibles – comme la médecine de la dépendance, individualisée et réductive, et la réduction radicale des méfaits.

Deuxièmement, cela représente une déchirante occasion manquée de nous mobiliser dans l’intérêt public. Imaginons le pouvoir qu’aurait eu un institut universitaire de plus de 500 membres sur une politique gouvernementale terriblement nocive, comme la fermeture de sites de consommation supervisée (Livingston, 2021), si au lieu de garder le silence, il avait promulgué une intervention communautaire orientée sur la justice sociale, qui devrait être au centre de toute démarche de santé publique.

La mesure dans laquelle les valeurs peuvent être reléguées en périphérie est illustrée par la dynamique qui accompagne une dépendance croissante à l’égard du financement philanthropique, lequel prend souvent la forme d’une transaction philanthropique privée liée à des secteurs que l’on sait être préjudiciables pour la santé, dont l’industrie des combustibles fossiles (Carroll, 2021; CPHA, 2015). D’après notre expérience, un tel financement a été jugé acceptable et n’a pas fait l’objet d’une consultation avec l’ensemble des membres, car il permettait le soutien de « l’excellence en recherche ».

Dans un article récent, Kothari et Smith (2022) décrivent deux pôles, avec un gradient entre les deux, pour caractériser les relations entre la santé publique et la politique. À l’un des deux pôles, la santé publique est vue comme étant apolitique, divorcée à dessein de toute réflexion et de tout engagement politique. Au pôle opposé, la santé publique se préoccupe de la santé du public (c’est-à-dire des contextes dans lesquels les gens naissent, grandissent, vivent, travaillent et vieillissent) et des inégalités béantes de pouvoir et d’argent qui conduisent à des iniquités structurelles dans les conditions préjudiciables à la santé.

Le premier pôle existe clairement (notre expérience n’en est qu’un exemple parmi d’autres), mais nous devons cesser de lui donner le même temps d’antenne que s’il s’agissait d’une version défendable de la santé publique. Une santé publique dépolitisée est fausse, préjudiciable et de plus en plus injustifiable dans le contexte du creusement des iniquités mis au jour par la crise des décès causés par les médicaments non réglementés et l’aggravation de la catastrophe climatique au Canada. La santé publique n’est pas, et ne peut pas être, moralement neutre. Tout institut qui ne le reconnaît pas et ne l’accepte pas explicitement cause du tort, tant au domaine qu’à la santé du public.

L’omission de notre institut d’épouser ces éléments et ces valeurs nous a poussées, deux titulaires de doctorats en santé publique qui avons consacré notre carrière à ce domaine et qui avons à nous deux joué de nombreux rôles, tissé de nombreux liens et apporté de nombreuses contributions à la santé publique, à démissionner.

Que cette mise en garde amène les communautés de la santé publique de nos établissements respectifs (universités, gouvernements et bailleurs de fonds) à faire ce qu’il faut pour « joindre le geste à la parole » en santé publique et nous donner ainsi les moyens de nous porter à la défense du meilleur de notre domaine.

Rebecca Haines-Saah, Professeure associée, University of Calgary

Lindsay McLaren, Professeure, University of Calgary; Rédactrice, RCSP
